# BK Channel in the Physiology and in the Cancer of Pancreatic Duct: Impact and Reliability of BK Openers

**DOI:** 10.3389/fphar.2022.906608

**Published:** 2022-05-24

**Authors:** Paolo Zuccolini, Paola Gavazzo, Michael Pusch

**Affiliations:** Institute of Biophysics, National Research Council, Genoa, Italy

**Keywords:** BK channel, pancreatic duct, pancreatic duct adenocarcinoma (PDAC), BK activators, cancer, NS11021, NS19504, BMS191011

## Abstract

BK (KCa 1.1, Slo-1) is a K^+^ channel characterized by an allosteric regulation of the gating mechanism by Ca^2+^ binding and voltage, and a high unitary conductance. The channel is expressed in many different tissues, where it is involved in the regulation or the fine-tuning of many physiological processes. Among other organs, BK is expressed in the pancreatic duct, a part of the gland important for the correct ionic composition of the pancreatic juice. Unfortunately, the pancreatic duct is also the site where one of the deadliest cancer types, the pancreatic duct adenocarcinoma (PDAC), develops. In the past years, it has been reported that continuous exposure of cancer cells to BK openers can have a significant impact on cell viability as well as on the ability to proliferate and migrate. Here, we first summarize the main BK channel properties and its roles in pancreatic duct physiology. Then we focus on the potential role of BK as a pharmacological target in PDAC. Moreover, we discuss how results obtained when employing BK activators on cancer cells can, in some cases, be misleading.

## Introduction

The large-conductance Ca^2+^- and voltage-activated K^+^ (BK) channel has been intensively studied for its important physiological roles and its peculiar features. The first evidence of a Ca^2+^-induced K^+^ conductance was found by Meech in *Aplysia californica* and *Helix aspersa* in the early 1970s (Meech, 1972; Meech and Standen, 1974; Bentzen et al., 2007). Almost a decade later, two distinct groups characterized BK single channel currents in excised membrane patches from bovine chromaffin and mouse muscle cells, respectively (Marty, 1981; Pallotta et al., 1981). The gene encoding the BK channel α subunit (*KCNMA1*) was cloned in the early 1990s from *Drosophila melanogaster* (Atkinson et al., 1991; Adelman et al., 1992) and *Mus musculus* (Butler et al., 1993). The BK pore-forming α subunit associates with β and γ subunits, which modulate channel properties and pharmacology; such functional diversity is further expanded by different splice variants and posttranslational modifications (Li and Yan, 2016; Latorre et al., 2017; Gonzalez-Perez and Lingle, 2019). BK channels are ubiquitously expressed in a large variety of excitable and non-excitable cells, where they play an important role in many physiological processes (Latorre et al., 2017; Kshatri et al., 2018; Sancho and Kyle, 2021).

PDAC is one of the deadliest types of cancer, given the lack of specific symptoms and resistance to chemotherapy (Fan et al., 2020; Zhang et al., 2020). BK has been found to be expressed both in the pancreatic duct epithelia cells (PDECs) (Gray et al., 1990; Hede et al., 2005; Venglovecz et al., 2011) and in PDAC cell lines (Zuccolini et al., 2022). Many studies in the literature report that sustained opening of the BK channel with activators reduces the ability to proliferate and migrate of different cancer cell lines. Unfortunately, according to new available data, this does not seem to be the case for Panc-1, a cell line derived from a primary PDAC.

## Main Text

### Brief Summary of the BK Channel Properties

BK displays a dual gating mechanism that regulates channel opening via depolarization and Ca^2+^ binding and is characterized by a high single channel conductance. Indeed, for both human recombinant and native channel, conductance values of 170 up to 230 pS are reported, which depend on the experimental conditions, for example symmetrical or physiological K^+^ concentrations (Ahring et al., 1997; Ransom and Sontheimer, 2001; Sandle et al., 2007). The complex interplay between the two gating processes became clearer thanks to recently published cryo-EM structures (Hite et al., 2017; Tao et al., 2017; Tao and MacKinnon, 2019). The channel architecture is similar to that of tetrameric voltage- and ligand-gated K^+^ channels, with a central pore surrounded by four voltage-sensing domains. Each BK subunit includes a voltage sensor domain formed by five transmembrane segments (VSD, S0-S4), a pore domain (PD, S5 and S6) and a cytosolic tail domain (CTD). BK channels are less sensitive to voltage compared to Shaker-like Kv channels, likely because the S4 segment has only three positively charged residues and a proper gating charge transfer center is lacking. The CTD comprises two regulators of K^+^ conductance (RCK) domains termed RCK1 and RCK2, and an additional regulatory site that can bind Mg^2+^ ions is located at the interface between the so-called gating ring and the transmembrane region (Shi et al., 2002; Xia et al., 2002; Yang et al., 2008).

Ca^2+^ binding and voltage sensor activation enhance channel opening by a dual-allosteric mechanism described in a Markov model (Horrigan and Aldrich, 2002) and confirmed by structures (Hite et al., 2017; Tao et al., 2017). Ca^2+^ binding to the CTD induces conformational changes that enhance pore transition to the open state. However, at (negative) resting membrane potentials, the S4 segment and the S4-S5 linker are in a conformation that negatively interferes with this mechanism. Moreover, as previously mentioned, the VSD is poorly voltage-sensitive. Therefore, significant changes in open probability -at low Ca^2+^ levels-are appreciable only at very positive voltages. Local increase in Ca^2+^ concentration favors the CTD conformational change to the bound form, which in turn exerts a displacement of the S4-S5 linker and pulls the S6, the segment that lines the pore. Therefore, an increase of intracellular calcium concentration ([Ca^2+^]_i_) would left-shift the channel voltage-dependence toward much less positive potentials.

As the channel is activated by calcium binding, it can exert a negative feedback mechanism on oscillations of [Ca^2+^]_i_. Such a mechanism is, for example, important for the regulation of the degree of tension in vascular and urinary smooth muscles, where local increases of [Ca^2+^]_i_ induce BK opening, which hyperpolarize the membrane potential deactivating Ca^2+^ channels (Brenner et al., 2000; Herrera et al., 2000). Another negative feedback mechanism has recently been found in neurons, where Ca^2+^ entry via NMDARs activates BK, inducing a membrane repolarization which, in turn, halts NMDAR activity (Zhang et al., 2018; Gomez et al., 2021). BK is also important for flow-induced K^+^ secretion and K^+^ homeostasis in the distal nephron as well as for the regulation of calcium oscillation in α- and β-cells of the pancreas (Rieg et al., 2007; Jacobson et al., 2010; Dickerson et al., 2019).

BK channel are sensitive to different activators and inhibitors (Bentzen et al., 2014). In the following paragraphs, we report examples for the employment of such molecules; a list of well-known BK openers and blockers mentioned in this mini-review is reported in Table 1.

**TABLE 1:** commonly used BK activators and inhibitors.

Activators	—	Blockers	—
NS1619	Olesen et al. (1994)	Paxilline	Knaus et al. (1994)
Phloretin	Koh et al. (1994)	Iberiotoxin	Galvez et al. (1990)
Pimaric acid	Imaizumi et al. (2002)	Charybdotoxin	Miller et al. (1985)
NS11021	Bentzen et al. (2007)	TEA	Blatz and Magleby (1984)
BMS191011	Romine et al. (2007)	—	—
NS19504	Nausch et al. (2014)	—	—
DHS-I	McManus et al. (1993)	—	—
Mallotoxin	Zakharov et al. (2005)	—	—

### BK Channels in the Pancreatic Duct Epithelial Cells

In exocrine pancreas electrolyte secretion is carried out mainly by PDECs which are particularly specialized in bicarbonate (HCO_3_^−^) secretion (Lee et al., 2012). The main physiological role of PDECs is indeed to secrete a HCO_3_^−^-rich fluid, which transports the enzymes to the duodenum and raises the pH of the small intestine in order to neutralize the acidic stomach content (Venglovecz et al., 2015). In the luminal membrane of PDECs, HCO_3_^−^ secretion is mediated by Cl^−^/HCO_3_^−^ exchangers, which work in concert with the CFTR Cl^−^ channel (Wang et al., 2006; Stewart et al., 2009). The efflux of HCO_3_^−^ is crucial for proper functioning of the pancreas, indeed insufficient ductal HCO_3_^−^ and fluid secretion can seriously damage the gland (Durie, 1989; Scheele et al., 1996). The BK channel is expressed in PDECs and has been found both on the basolateral as well as the luminal membrane (Gray et al., 1990; Hede et al., 2005; Venglovecz et al., 2011). The localization of the channel within ductal cells seems to vary between the two most studied animal models, namely rat and guinea pigs (Hayashi and Novak, 2013). In PDECs, BK is believed to contribute to creating the proper driving force for HCO_3_^−^ secretion from the luminal membrane (Gray et al., 1990; Venglovecz et al., 2011; Lee et al., 2012) given its large conductance for K^+^ ions: cell-attached measurements with K^+^-rich pipette solution in isolated rat PDECs revealed a conductance of ∼170 pS (Hayashi et al., 2012). Expression levels of the four BK β subunits in the rat pancreatic tissue were estimated by RT-PCR in 2005 by Hede and others, who found that only β1 was expressed (Hede et al., 2005).

In the early 1990s it was proposed that the channel is involved in the secretion increase in response to secretagogues like secretin via a cAMP/PKA signaling pathway (Gray et al., 1990). The secretin receptor is coupled to adenylyl cyclase; therefore, its activation increases cAMP concentration (Folsch et al., 1980; de Ondarza and Hootman, 1995). Gray and others found that basolateral stimulation of rat PDECs with secretin induced a Ca^2+^-independent increase of the channel open probability, which remained in excised patches (Gray et al., 1990). Further experiments of the same study showed that channel activity is increased by phosphorylation of the channel or regulatory subunits (possibly β1, the only β subunit expressed in the tissue) by PKA, suggesting that, under physiological conditions, BK channels play an essential role in cAMP-stimulated HCO_3_^−^ secretion. However, years later, it was observed that in guinea pig ducts the BK specific blocker iberiotoxin did not alter bicarbonate secretion induced by secretin, suggesting that BK is not involved in the stimulatory effect of the hormone in this animal model (Venglovecz et al., 2011). Moreover, it has been reported that channel response to PKA phosphorylation depends on the splice-variant of the α-subunit: the ZERO variant is activated by PKA, which conversely inhibits another variant named STREX-1 (Tian et al., 2001). Nevertheless, BK is probably important for bicarbonate secretion, as the luminal application of the channel opener NS11021 induces an increase of HCO_3_^−^ release (Venglovecz et al., 2011).

BK-assisted HCO_3_^−^ efflux was found to be important for PDECs first response to low concentrations of bile acid in the duct, in the initial phases of a gallstone-induced pancreatitis. It is indeed reported that low luminal doses of one of the primary bile acids, chenodeoxycholate (CDC), induce an increase of HCO_3_^−^ secretion associated with an elevation of [Ca^2+^]_i_ (Venglovecz et al., 2008). In 2010, Venglovecz and others identified in BK the apical Ca^2+^-dependent channel responsible for the observed phenomenon (Venglovecz et al., 2011). The authors suggest that opening of BK hyperpolarizes the cell membrane, increasing the driving force for anion secretion.

In addition to the above-mentioned studies, it is also worth to mention that BK is thought to be involved in the modulation of the secretion induced by ATP and UTP binding to purinergic receptors P2Y, although the mechanism in not completely clear (Hede et al., 1999; Hede et al., 2005; Ha and Cheong, 2017). PDECs express G-protein coupled P2Y and ligand-gated ion channel P2X receptors; it has been observed that the perfusion of ATP/UTP on the basolateral side reduced fluid and HCO_3_^−^ secretion (Ishiguro et al., 1999). In this context, stimulation of P2Y receptors was shown to decrease K^+^ conductance in PDECs, which would in turn slow down HCO_3_^−^ secretion (Hede et al., 1999). Further experiments with heterologously expressed P2Y2 and BK demonstrated that activation of the purinergic receptor is able to inhibit BK, providing an explanation for the basolateral ATP/UTP-induced decrease in HCO_3_^−^ secretion (Hede et al., 2005). However, the picture turned out to be more complex as the activation of P2Y_4_ (also present in PDECs) resulted in an increase of BK current, and both P2Y_4_ and P2Y_2_ were able to activate another Ca^2+^-gated K^+^ channels, i.e. the intermediate conductance (IK) channel, also expressed in PDECs (Hede et al., 2005). Therefore, it is not clear what is the specific contribution of BK in the response to ATP and UTP.

### Sustained BK Activation Could Have a Negative Impact on Proliferation, Migration and Viability of Different Types of Cancer Cell Lines but Not PDAC

In the past years ion channels have emerged as important actors for tumor biology (Lastraioli et al., 2015). As mentioned above, BK opening can induce a strong hyperpolarization in the membrane potential. Alterations in the membrane voltage have been proposed to be extremely important in controlling the cell cycle (Abdul Kadir et al., 2018); this suggests that BK can play a key role in cancer and indeed; many studies in the literature show that the activation of BK can have a significant impact on cancer cell proliferation and migration.

For example, it has been observed that BK opening can reduce the migration of glioma cells. Bordey and others reported that prolonged exposure to acetylcholine (Ach) and muscarine led to a rise of [Ca^2+^]_i_ and stopped the migration of astrocytoma cells U373MG (Bordey et al., 2000). Further investigation showed that the increase of [Ca^2+^]_i_ was followed by BK activation. It remained nevertheless unclear if the inhibition of cell migration was directly due to BK channel gating per se or if the cause was the increase of Ca^2+^ or other second messenger signals. Few years later, Kraft et al. suggested a significant impact of BK activation on the migration of glioma cells (Kraft et al., 2003). They found that BK activators phloretin and NS1619, as well as ACh, were able to reduce cell migration velocity by approximately 50% and that the latter effect could be reversed by BK blockers.

Moreover, it has been reported that BK openers induce U251 glioma and small cell lung cancer (SCLC) cells to swell (Hoa et al., 2007; Hoa et al., 2014). Also, the opening of BK has been found to induce a decrease in the viability and the migration of breast cancer cells. In a recent work, Sizemore and collaborators treated MDA-MB-231 cells with two distinct BK activators: BMS-191011 and NS-11021. The latter were able to induce a strong membrane hyperpolarization and, after prolonged treatment, also a reduction of live cells and migration (Sizemore et al., 2020). Moreover, low doses of the BK agonist BMS-191011 triggered apoptosis in MDA-MB-231 cells, where cleavage of procaspase three into caspase three was observed. In another work, Han et al., demonstrated that the BK activator NS1619 is able to inhibit proliferation and to induce apoptosis in ovarian cancer cells (Han et al., 2008).

Our group has recently investigated the effect of different BK openers on PDAC cells, which had not yet been exhaustively evaluated for this type of cancer (Remigante et al., 2021). We tested the impact on Panc-1 cell viability, migration and proliferation of two of the newest and most effective known BK activators, namely NS11021 and NS19504.

It has been reported for other types of cancers that the neoplastic tissue can differ from the normal one in terms of BK splice variants and regulatory subunits expression. For example, Egland et al. observed in pancreatic cancer tissues samples the expression of the γ1 protein LRRC26 (also known as CAPC), which left-shifts the voltage-dependence of the channel activation to more hyperpolarized voltages (Egland et al., 2006; Gonzalez-Perez et al., 2022). For the parotid BK channel V_1/2_ values of 50 mV have been measured when the channel was co-expressed in CHO cells with the LRRC26 protein and of 189 mV for the α subunit expressed alone (Almassy and Begenisich, 2012). Moreover, Liu et al. reported the presence of the splice variant gBK in a pancreatic adenocarcinoma sample (Liu et al., 2002). This splice variant is characterized by a normal BK unitary conductance of ∼250 pS but displays slower activation and higher sensitivity to [Ca^2+^]_i_ than its closest homolog hbr5 (Liu et al., 2002). The endogenous currents displayed by Panc-1 cells at low [Ca^2+^]_i_ (∼20 nM) are characterized by a deviation from linearity and a sudden increase of current density at voltages ≥ +80 mV, which are likely due to the activation of BK channels (Zuccolini et al., 2022). No clear evidence of LRRC26-mediated strong left shift of BK voltage-dependence can be observed in this cell line. This is in agreement with what has been reported by gene expression analysis (see (Klijn et al., 2015), EGA: EGAS00001000610).

Both NS11021 and NS19504 are able to induce a Paxilline-sensitive outward current in Panc-1 cells, which maintains a certain degree of voltage-dependency. We then tested the long-term effects of the two molecules on Panc-1 cell viability (72 h exposure). NS11021 decreased cell viability within 72 h. However, several lines of evidence raised serious doubts that this effect is dependent on BK activation. First, another cancer cell line (melanoma IGR37) displayed a similar viability drop after a 72 hours-long treatment with NS11021 even though the expression of the channel is ∼10^4^ times lower in IGR37 cells than in Panc-1 cells (Ferrera et al., 2021; Zuccolini et al., 2022), and no BK like currents were activated by NS11021 in IGR37 cells (Remigante et al., 2021). Moreover, the other tested BK activator, NS19504, did not have any impact on Panc-1 viability in the same experimental conditions. Subsequently, we investigated the effect of the activators on Panc-1 migration ability with wound healing assays: neither NS11021 nor NS19504 induced a reduction in cell migration with respect to untreated Panc-1 cells. Data from real-time cell proliferation experiments were similar to those from cell viability assays: NS19504 did not have any effect, while NS11021 slowed down the proliferation of Panc-1 cells but also of the negative control IGR37. Most importantly, imaging experiments revealed that both molecules induced an increase of [Ca^2+^]_i_. In the case of NS19504, the calcium increase is partially correlated with BK activation as Paxilline strongly reduced the raise in [Ca^2+^]_i_. Conversely, NS11021 is able to activate a calcium conductance within the cell membrane in a BK-independent manner. This could explain why the molecule affected also the negative control IGR37 cells, where the channel is poorly present. Surprisingly, when we tested the ability of BMS191011, another reportedly specific BK opener (Table 1, Romine et al., 2007), to elicit BK current in Panc-1 cells we found that the compound was much less efficient than NS11021 and NS19504. Induced currents at +120 mV compared to those measured in standard bath solution were 14 times higher for NS11021, 7 times higher for NS19504 while only 2 times higher for BMS19504.

According to these recent data, it seems that the activation of the BK channel is not a good strategy for slowing down the survival and carcinogenicity of PDAC cells, although it has been suggested to be efficient for other cancer cell types. Indeed, even the more specific BK activator NS19504 did not affect viability nor proliferation or migration of Panc-1 cells. Moreover, the activation of BK can induce, in this cell type, an increase in [Ca^2+^]_i_ which is a signal related to many aspects of cellular physiology.

## Discussion

BK is a ubiquitously expressed channel which has attracted scientists’ attention for decades. Because of its peculiar calcium- and voltage-dependent dual gating mechanism and its large conductance, this channel plays a role in many physiological pathways involving membrane hyperpolarization. Among the different tissues, BK is expressed in the epithelial cells of the pancreatic duct. Here, it has been proposed to contribute in different ways to bicarbonate efflux especially in the presence of bile acid in the duct. A deep understanding of the BK involvement in the molecular pathways underlying the HCO3^-^ secretion regulation appears problematic. Different observations made in different animal models or focused on different cell compartments, might appear contradictory. Given the large number of channels and transporters expressed in PDECs, their specific localization in the luminal vs. apical membrane, their complex interactions and the different external stimuli that can modulate them it can actually become complicated to put together the various pieces of information in a general scheme in which the BK function is completely defined. In this regard, to get an idea of the complexity of the system, we recommend specific reviews: (Lee et al., 2012; Hayashi and Novak, 2013; Venglovecz et al., 2015; Schnipper et al., 2020; Venglovecz et al., 2021). The channel is expressed also in the neoplastic formation that originates in this fundamental part of the digestive system, the PDAC.

### Considerations About Cell Lines and Relative BK Channel Diversity

Studies in literature report that the prolonged application of various BK channel openers decreases viability and migration in different cancer cell lines. Unfortunately, recent experiments from our laboratory revealed that prolonged treatment with BK activators does not have such effects on PDAC cells, namely Panc-1. Indeed, Panc-1 cells exposed to different types of openers did not show any alteration neither in viability, nor in proliferation or migration. Moreover, similar results were obtained also with the melanoma line IGR39. It is important to highlight that the impact of BK activation on membrane voltage (and in turn cells duplication) may differ between different cell lines. Indeed, the gating range of the BK channel can considerably vary between different cell types, which might express different BK splice variants and/or auxiliary subunits and might have different basal levels of [Ca^2+^]_i_. For example, BMS191011 induced membrane hyperpolarization in breast cancer cells (Sizemore et al., 2020). However, Sizemore and collaborators did not test if BMS191011 actually activated BK in the breast cancer cells. This is relevant in that our group has recently found that the compound only poorly activated the BK channel in PDAC and melanoma lines (Remigante et al., 2021). A possible explanation for the divergent effects of BK agonists between Panc-1 and other cancer cells, could be that the basal Ca^2+^ concentration in Panc-1 cells [around 200 nM (Remigante et al., 2019; Zuccolini et al., 2022)] is not high enough to reach significant channel activation at negative voltages even with the activator present because the gating range is too right-shifted. Nevertheless, it can be hypothesized that, during the constitutive presence of the activators, local spatiotemporal fluctuations can increase the concentration of calcium in certain cell compartments rather than in the entire cytoplasm. In addition, since membrane potential and calcium concentration vary in critical points of the cell cycle, the action of the activators can occur only in those key moments, but is still enough to overall slow down cancer cell growth. Another issue to be considered is that regulatory subunits can affect BK response to activators. McManus and others observed that the channel α subunit was insensitive to the agonist DHS-I (McManus et al., 1993; Giangiacomo et al., 1998) when expressed alone in *Xenopus* oocytes, while the activator worked when α and β subunits were co-expressed in the heterologous system (McManus et al., 1995). Conversely, we recently discovered that the VRAC blocker DCPIB is able to activate the BK channel even when the α subunit is expressed alone in HEK293 cells (Zuccolini et al., 2022). The action of another BK agonist, Mallotoxin (MTX), is not affected by β subunits (Zakharov et al., 2005). Nevertheless, in the presence of the above-mentioned LRRC26 protein, which moves the activation voltage window to less negative potentials, MTX does not induce any further left-shift of the voltage dependence (Almassy and Begenisich, 2012). It seems therefore that a deep understanding of the channel composition in the neoplastic tissue of interest is crucial for designing and planning experiments with positive outcome. For example, it would be important to determine if and how much LRRC26 is expressed in the analyzed cancer cell line, based on available publications (Egland et al., 2006) and databases like the Cancer Cell Lines Encyclopedia (CCLE, https://ctd2-data.nci.nih.gov/Public/TGen/CCLE_RNA-seq_Analysis/). According to CCLE, the LRRC26 γ1 regulatory subunit seems to be poorly expressed in the cancer lines on which BK activators had been shown to have anti-cancer effects, although it could be detected by RT-qPCR in MDA-MB-231 cells (Egland et al., 2006). However, unfortunately, not all the studies in which BK activators are employed on cancer cells provide detailed information about the cells resting state and the expression of regulatory proteins. This might be a major problem when one approaches the study of the potentiality of BK as a target for cancer treatment.

### Brief Discussion About BK Activators Differences, Efficacy and Reliability

Another point we would like to stress is that BK activators should be used with caution, as some of them may have side effects on other membrane proteins. For example, NS1619 had been thought for years to be a specific and potent activator of the BK channel, enhancing the open probability by interacting with the channel from the intracellular side (Gessner et al., 2012; Malysz et al., 2013). Unfortunately, it has been reported that NS1619 can induce Ca^2+^ release in pig smooth muscle cells (Yamamura et al., 2001). Other experiments by Wrzosek showed that NS1619 directly affects SERCA activity in sarcoplasmic reticulum vesicles, increasing Ca^2+^ leakage from isolated vesicles (Wrzosek, 2014). Another known side effect of this molecule is the blockade of the intermediate conductance calcium-gated K^+^ channel KCa3.1 (Cai et al., 1998). NS1619 has also been reported to affect the gating of L-type Ca^2+^ channels and Ca^2+^-gated Cl^−^ channels (Park et al., 2007; Saleh et al., 2007). NS11021 is a potent “newer generation” BK activator, able to increase the open probability of the channel by altering gating kinetics without affecting the single-channel conductance (Bentzen et al., 2007). The compound seemed to have a direct and Ca^2+^-independent action on BK, as Rockman et al. reported that it could increase the open probability of a truncated channel lacking the CTD (Rockman et al., 2020). Nevertheless, in Panc-1 and IGR39 cancer cell lines, in addition to directly activating BK, the compound activated a Ca^2+^ conductance leading to intracellular calcium increase (Remigante et al., 2021). Among the BK activators, NS19504 appears to be the most specific. It has been tested by radioligand binding assays against 68 different channels and receptors (Nausch et al., 2014). Effects were observed only against norepinephrine transporter (SLC6A2), dopamine transporter (SLC6A3), and sigma nonopioid intracellular receptor 1 (σ1R), while the other 65 channels and receptors were not affected (Nausch et al., 2014). It is worth to specify that the molecular mechanisms by which activators modulate channel opening can be very diverse. While some of them bind the channel from the intracellular side, like NS11021 or NS1619, (Olesen et al., 1994; Bentzen et al., 2007), others like MTX act from the outside (Zakharov et al., 2005; Wu et al., 2007). It was indeed reported that in whole-cell experiments MTX affected channel gating when perfused from the outside but not when applied to the cytosol via patch pipette (Zakharov et al., 2005). Similarly, also DCPIB binds to the extracellular side of the channel, increasing the open probability (Zuccolini et al., 2022).

### Final Considerations

Another critical point in using BK activators in Panc-1 is the fact that, in this cell type, BK opening itself might generate an increase of [Ca^2+^]_i_, possibly by creating the electrical driving force for calcium entrance. For this reason it would be hard to understand if effects observed after treatments with BK activators are solely dependent on their action on BK channels or rather due to the increase of intracellular [Ca^2+^]_i_. Finally, the principle according to which membrane hyperpolarization slows down cancer growth has been proposed to work for some cancer cell types but, in contrast, other studies report anti-cancer action for BK blockers (Goda et al., 2018; Li et al., 2018; Noda et al., 2020).

We therefore conclude that the employment of BK activators presents quite some issues to which scientists should pay attention. In this regard, it appears important that studies in which BK activators are used on cancer cells, investigate several parameters that are relevant for a proper interpretation of effects on viability, migration and proliferation. In particular, basal cellular conditions [resting potential, resting (Ca^2+^)_i_, BK splice variants, BK subunit expression] need to be assessed and the biophysical properties of the native BK current under these conditions need to be determined. Furthermore, the employed molecules need to be tested for side-effects on other proteins and, in particular, on unspecific increases of intracellular calcium. In the absence of this information, the outcome of administration of BK openers is hard to interpret, as one cannot distinguish whether a given effect is due to the specific activation of BK or is the result of off-target effects. Such a variety in the impact of BK agonists and blockers does not necessarily question the results reported in the literature nor preclude the importance of these molecules in specific cases, but it can raise an alarm flag regarding the use of presumably specific BK channel modulators as anti-cancer agents.
